# Variability of estimated glomerular filtration rate and ^99m^Tc-DTPA glomerular filtration rate: implications for a single time-point sampling regime

**DOI:** 10.1097/MNM.0000000000001674

**Published:** 2023-02-27

**Authors:** Lara M. Bonney, Daniel R. McGowan

**Affiliations:** a Department of Medical Physics and Clinical Engineering, Churchill Hospital, Oxford University Hospitals NHS Foundation Trust; b Department of Oncology, University of Oxford, Oxford, UK

**Keywords:** estimated glomerular filtration rate, glomerular filtration rate, single sample, ^99m^Tc-diethylene–triamine–pentaacetate

## Abstract

This work aimed to determine the implications of the variability in estimated glomerular filtration rate (eGFR) for the prediction of measured GFR (mGFR) for selection of sampling time-point in single-sample ^99m^Tc-diethylene–triamine–pentaacetate (DTPA) mGFR.

**Methods:**

Patient studies were used to compare eGFR and mGFR (*n* = 282). The eGFR was calculated using the Chronic Kidney Disease Epidemiology Collaboration 2009 equation, from serum creatinine values measured in the laboratory (*n* = 27) or using a point-of-care testing device (*n* = 255). The mGFR was taken as the true value, and the root mean square error (RMS_err_) in eGFR was calculated. Receiver operator characteristic curves were generated comparing the sensitivity and specificity of eGFR for the prediction of mGFR within the British Nuclear Medicine Society (BNMS) 2018 guideline ranges.

**Results:**

The overall eGFR RMS_err_ was 19.3 mL/min/1.73 m^2^. Use of eGFR to predict mGFR in the ranges specified in the BNMS 2018 guidelines (25–50; 50–70; 70–100; and >100) achieved the following specificity and sensitivity for each individual range (97%, 71%; 92%, 47%; 81%, 48%; and 74%, 90%). For the middle ranges (50–70 and 70–100) the sensitivity is very low, less than 50%; more studies are classified incorrectly on the basis of eGFR in these ranges than correctly.

**Conclusion:**

This work shows that serum creatinine eGFR is not sufficiently accurate to predict the optimum single-sample time-point for ^99m^Tc-DTPA mGFR prior to measurement. It is the recommendation of this study that a single sampling time-point should be chosen for studies eGFR > 40 ml/min/1.73 m^2^ as opposed to the use of eGFR to determine the sampling time-point.

## Introduction

In 2018, the British Nuclear Medicine Society (BNMS) released new guidelines on the measurement of glomerular filtration rate (GFR) using plasma sampling. These guidelines recommend the use of a single-sample technique for adult patients without ascites, oedema or other condition that could cause variation in the volume of distribution [1]. This is a significant change from the previous 2004 guidelines which recommended the multi-sample slope-intercept technique [2–4]. The single-sample technique has been shown to be accurate as compared to a multi-sample slope-intercept GFR [5,6] and has obvious benefits for patients and nuclear medicine departments, such as improved patient comfort due to fewer blood samples and resource savings. However, the mathematical optimum sampling time-point for single-sample studies is dependent on GFR [3–5]. Therefore, the BNMS guidelines recommend the sample collection time is chosen based on the expected measured GFR (mGFR) estimated from a serum creatinine GFR measurement [estimated GFR (eGFR)]. BNMS 2018 guidelines recommend five different sample time-points, dependent on patient-expected GFR [1]. A recent multi-centre study by McMeekin *et al.* (2022) calculated the error on the single-sample mGFR measurement as compared to the slope-intercept mGFR measurement and based on the minimum error recommended that sample time should be chosen from four ranges instead of five [7].

If there were no variation between eGFR and measured glomerular filtration rate (mGFR) then mGFR studies would not be performed. Thus, errors in the eGFR measurement to predict mGFR must be expected. Previous work by Hutton *et al.* (using ^51^Cr-EDTA) considered the ability of eGFR to predict mGFR at low GFR values. Variation between eGFR and the mGFR was demonstrated and the study found that to ensure all patients with GFR <25 mL/min/1.73 m^2^ were identified an eGFR threshold of <40 mL/min/1.73 m^2^ was required [8]. This demonstrates the possible errors in sampling time that could occur due to eGFR variability. This work looks at a wider GFR range measured from ^99m^Tc-diethylene–triamine–pentaacetate (DTPA) and investigated the implication of variability in eGFR on its use for prediction of mGFR range and sampling time-point for single-sample mGFR studies.

## Methods

### Dataset

An audit was completed of 307 adult patients who underwent a GFR examination at Oxford University Hospitals, NHS Foundation Trust between March 2019 and September 2022. These are all studies completed following the move to a ^99m^Tc-DTPA service, from ^51^Cr-EDTA. For inclusion studies required a sample time within 20 min of specified sampling time and eGFR measurement within 28 days of mGFR. One study was excluded as the serum creatinine measurement was not recorded, six studies were excluded as the sample time was more than 20 min outside the time specified in the protocol and one study was excluded as the eGFR measurement had been performed 31 days prior to the GFR study, compliant with local protocol which specifies 1 month but outside the 28-day limit applied here in line with other work in the field [7,8]. Studies with large errors associated with the estimation of body surface area (BSA) (amputees) or volume of distribution (ascites/oedema) or both were also excluded (*n* = 17). The final dataset comprised 282 patients with an age range of 18–89 years of age with a mean of 52, and a close to equal split of male and female patients (144 and 138). Two hundred sixty patients had a single-sample GFR study and 22 patients underwent a multiple-sample slope-intercept study as per local protocol.

### Sample collection

Patient eGFR was determined from a serum creatinine measurement either from previous laboratory blood work (*n* = 27) or point-of-care (POC) testing (*n* = 255) using an i-STAT device (Abbott Point of Care Inc., Princeton, New Jersey, USA). The same i-STAT device was used throughout all POC testing measurements in this work. Although a difference between laboratory and POC testing results is expected, a systematic review of device performance found such biases in performance were consistent and predictable [9]. It is noted POC testing results have the added benefit of occurring only a few hours prior to the mGFR measurement, as compared to laboratory measurements which typically occur at least one day prior. POC testing eGFR measurements can therefore be considered contemporaneous with mGFR. The 2009 Chronic Kidney Disease Epidemiology Collaboration (CKD-EPI) equation was used to calculate eGFR from serum creatinine [9,10]. The equation was applied to laboratory and POC testing serum creatinine measurements. In line with recommendations by Gansevoort *et al.*, the 2009 CKD-EPI equation was used with no race correction factor applied, as is common practice across most European countries [11].

The patients were administered 10 ± 1 MBq ^99m^Tc-DTPA intravenously. Following previous work, for patients with eGFR of ≤40 mL/min/1.73 m^2^ a three-sample slope-intercept method was followed with samples at 120, 180 and 240 min (*n* = 13). This is as per local protocol to ensure all patients with an mGFR ≤ 25 mL/min/1.73 m^2^ have a slope-intercept GFR rather than single-sample GFR [8]. The three-sample slope-intercept method was followed for an additional nine patients for other clinical indications (e.g. known inaccuracy of eGFR due to high muscle mass or cachexia in line with BNMS guidelines [1]). For all other patients with eGFR > 40 mL/min/1.73 m^2^ a single sample was collected at 240 min (*n* = 260). Samples were spun in a centrifuge at 1000*g* for 5 min to separate plasma. A standard was prepared each day for the measurement of system sensitivity. Two 1 mL samples of plasma were pipetted from each blood sample and counted alongside four standard samples in a Wallac Wizard 2470 (Perkin Elmer, Beaconsfield, Buckinghamshire, UK).

### Analysis

Throughout this work, mGFR is taken to be the true value of patient GFR. First, the absolute difference in the eGFR measurement compared to the mGFR measurement (Δ) is considered, Eq. (1). The root mean square error, RMS_err_, has been defined, for sample size *N*, as per Eqn. (2) with the root mean square error on the error, $RM{S_{err}}_{{}_{err}}$, defined as in Eqn. (4).


$$\begin{array}{l} \begin{array}{l} \Delta_{i}=GFR_{i}-eGFR_{i} \end{array} \end{array}$$ (1)

$$\begin{array}{l} \begin{array}{l} RMS_{err}=\sqrt{\frac{\overset{N}{\underset{i=0}{\sum }}{\Delta_{i}}^{2}}{N}} \end{array} \end{array}$$ (2)

$$\begin{array}{l} \begin{array}{l} \tilde{\Delta }=\frac{\overset{N}{\underset{i=0}{\sum }}\Delta_{i}}{N} \end{array} \end{array}$$ (3)

$$\begin{array}{l} \begin{array}{l} RM{S_{err}}_{{}_{err}}=\sqrt{\frac{\overset{N}{\underset{i=0}{\sum }}{\left(\Delta_{i}-\tilde{\Delta }\right)}^{2}}{N}} \end{array} \end{array}$$ (4)

**Fig. 1:**
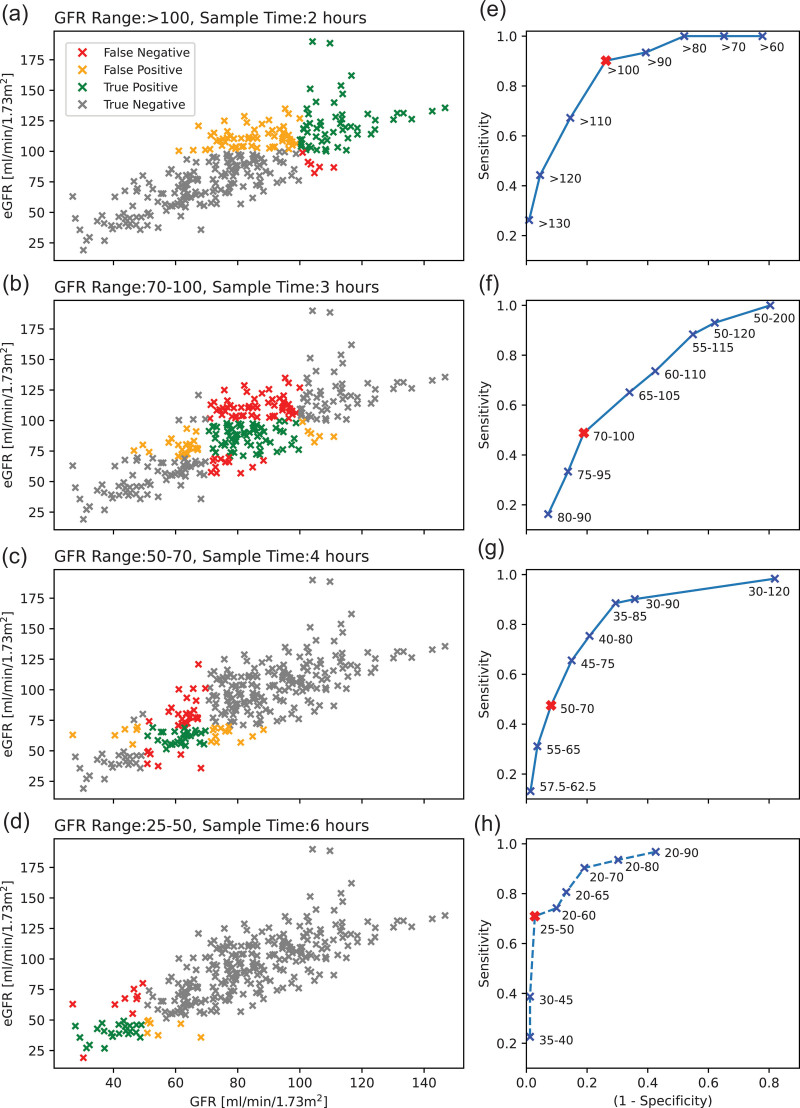
BNMS 2018 Guideline ranges. (a–d) eGFR plotted against mGFR with each datapoint colour coded with whether it corresponds to a True Positive, True Negative, False-Positive or False Negative case when using an eGFR range equal to the desired mGFR range for prediction. (e–h) Receiver operator characteristic curves for the ability of eGFR to predict a specified mGFR range. The point at which the eGFR range equals the mGFR range is highlighted in red. The sensitivity and specificity at this point are what is achieved when eGFR is taken as a perfect predictor of mGFR. (d and h) The receiver operator characteristic curve for the GFR range 25–50 is shown as a dashed line due to a low number of GFR datapoints to define the sensitivity and specificity in this range. BNMS, British Nuclear Medicine Society; eGFR, estimated glomerular filtration rate; mGFR, measured glomerular filtration rate.

**Fig. 2:**
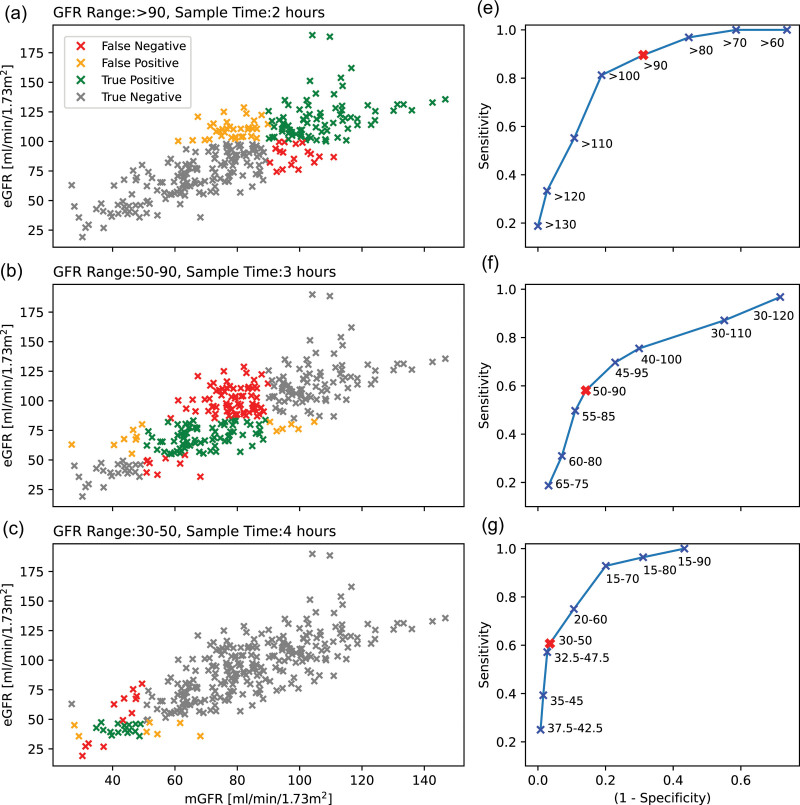
McMeekin *et al.* (2022) ranges. (a–d) eGFR plotted against mGFR with each datapoint colour coded with whether it corresponds to a True Positive, True Negative, False Positive or False Negative case when using an eGFR range equal to the desired mGFR range for prediction. (e–h) Receiver operator characteristic curves for the ability of eGFR to predict a specified mGFR range. The point at which the eGFR range equals the mGFR range is highlighted in red. The sensitivity and specificity at this point are what is achieved when eGFR is taken as a perfect predictor of mGFR.

The sensitivity and specificity of eGFR for the prediction of mGFR are also considered. The sensitivity and specificity are calculated as per Eqn. (5) and (6). The width of the predictor eGFR range is varied to calculate a receiver operator characteristic curve for each specified mGFR range. The analysis will focus on maximising sensitivity. The following definitions are used in the calculations, this is also displayed visually in the left-hand columns of Figs. 1 and 2. True positive: eGFR range correctly predicts an mGFR within the specified range. True negative: eGFR range correctly predicts an mGFR outside the specified range. False positive: eGFR range incorrectly predicts an mGFR within the specified range. False negative: eGFR range incorrectly predicts the mGFR will fall outside the specified range.

$$\begin{array}{l} \begin{array}{l} Sensitivity=\frac{TruePositives}{TruePositives+FalseNegatives} \end{array} \end{array}$$ (5)

$$\begin{array}{l} \begin{array}{l} Specif\;icity=\frac{TrueNegatives}{TrueNegatives+FalsePositives} \end{array} \end{array}$$ (6)

## Results

**Fig. 3:**
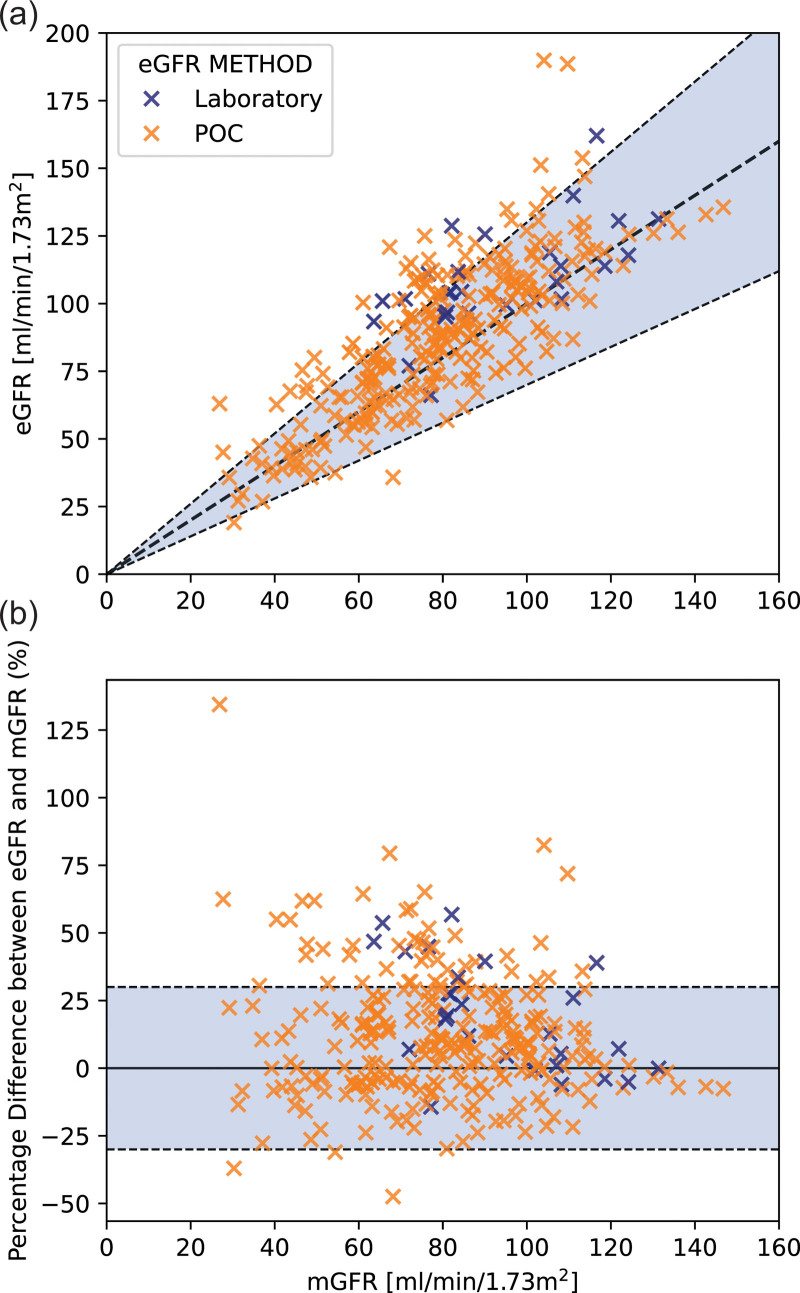
(a) Full dataset for eGFR and mGFR. The blue-shaded region highlights the area in which eGFR is within 30% of mGFR. (b) Percentage difference between eGFR and mGFR plotted against mGFR. It can be seen the data are positively distributed but appears relatively uniform in distribution with no significant trends. eGFR, estimated glomerular filtration rate; mGFR, measured glomerular filtration rate.

Figure 3a shows eGFR plotted against mGFR, the blue-shaded region highlights the area in which eGFR agrees with mGFR within 30%, 80.1% of datapoints are within this region. The shaded region is repeated in Fig. 3b which shows the percentage difference between eGFR and mGFR. Figure 3 shows on average the eGFR overestimated mGFR with a median percentage difference of 9.3%. The sample size for laboratory measurements is much smaller than that for POC testing measurements. There is no stark visual difference in the distribution of measurements for POC and laboratory observed in Fig. 3. The mean difference between eGFR and mGFR for POC testing was 11.0% (SD 23.1%), and for laboratory 21.4% (SD 20.2%).

**Fig. 4:**
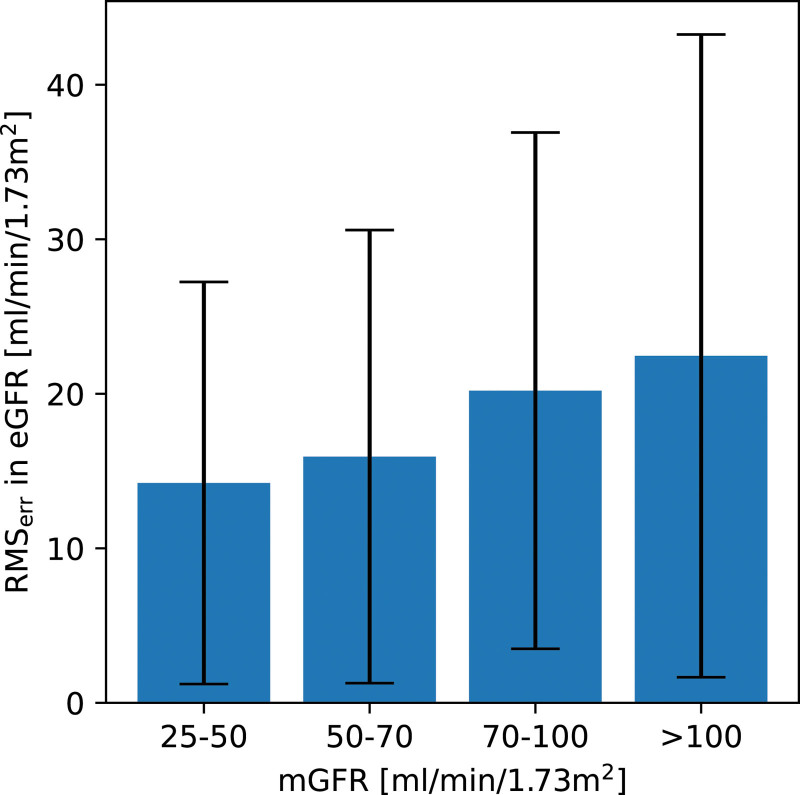
Variation in RMS_err_ in eGFR for the different mGFR BNMS 2018 guideline ranges. RMS_err_ is constant within error across the ranges. BNMS, British Nuclear Medicine Society; eGFR, estimated glomerular filtration rate; mGFR, measured glomerular filtration rate; RMS_err_, root mean square error.

The Previously defined. (RMS_err_) for the total dataset was found to be 19.3 ± 17.1 mL/min/1.73 m^2^. Table 1 shows the RMS_err_ on eGFR for the full range of mGFR values and the variation for different mGFR ranges. The same calculations were repeated for the BNMS 2018 guideline ranges as shown in Fig. 4. This shows the absolute RMS_err_ is constant to within error across the range of mGFR values measured.

**Table 1:** Root mean square error and the error on the root mean square error for varying measured glomerular filtration rate ranges

mGFR range (mL/min/1.73 m^2^)	*N*	$RMS_{err}$ (mL/min/1.73 m^2^)	$RM{S_{err}}_{{}_{err}}$ (mL/min/1.73 m^2^)
<40	12	13.3	12.6
40–50	19	14.8	13.2
50–60	19	12.5	11.8
60–70	42	17.3	15.7
70–80	52	21.8	16.8
80–90	42	19.8	17.1
90–100	35	18.2	15.6
100–110	33	26.3	23.4
110–120	18	20.3	18.1
>120	10	7.3	5.9

mGFR, measured glomerular filtration rate; $RMS_{err}$, root mean square error; $RM{S_{err}}_{{}_{err}}$, root mean square error on the error.

Receiver operator characteristic curves for eGFR as a predictor of mGFR for each range specified in the BNMS 2018 guidelines are plotted in the right-hand columns of Figs. 1 and 2 for the BNMS 2018 guideline ranges and the ranges proposed by McMeekin *et al.* (2022), respectively. The width of the eGFR window has been varied, with the position centred on the mGFR specified range. As the eGFR width increases the window ceases to be symmetric on the mGFR specified range, as the limits of the data range are reached. The dataset is limited for low mGFR, with sensitivity and specificity poorly defined in the 25–50 mL/min/1.73 m^2^ and 30–50 mL/min/1.73 m^2^ ranges for the BNMS 2018 guidelines and McMeekin *et al.* (2022) ranges, respectively.

**Fig. 5:**
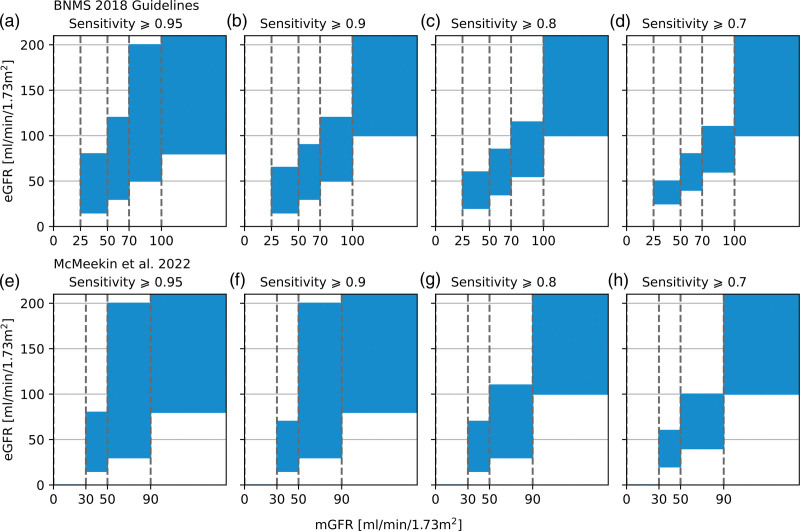
Each plot in this array shows a visual representation of the eGFR range required to achieve the target sensitivity for the specified mGFR range. Vertical overlap between adjacent blue boxes means the target sensitivity cannot be achieved in each individual mGFR range with uniquely identified eGFR ranges. (a–d) BNMS 2018 guideline ranges, (e–h) McMeekin *et al.* (2022) ranges. BNMS, British Nuclear Medicine Society; eGFR, estimated glomerular filtration rate; mGFR, measured glomerular filtration rate.

Figure 5 visualises the eGFR ranges required to achieve target sensitivity for each individual mGFR range. As the target sensitivity decreases from left to right, the degree of overlap between eGFR ranges decreases. There is still significant overlap between the eGFR ranges required to achieve a target sensitivity of 0.7 for each individual mGFR range.

## Discussion

This work aimed to determine whether it is feasible to predict GFR using serum creatinine eGFR for the purpose of improving the accuracy of single-sample mGFR. This is dependent on the variability of eGFR as compared to mGFR. High variability was found in eGFR with a median difference from mGFR of 9.3%, range −47.5% to 134.4%. The overall RMS_err_ on eGFR is large (19.3 mL/min/1.73 m^2^) in comparison to the size of the BNMS 2018 guideline ranges or those proposed by McMeekin *et al.* (2022). Considering the 2018 guideline ranges if a patient had an eGFR of 60 mL/min/1.73 m^2^ from the measured RMS_err_ 17.3 mL/min/1.73 m^2^ there are three potential BNMS single-sample time-points within error.

The high variability in eGFR translates into poor sensitivity and specificity for the prediction of individual mGFR ranges. To obtain a high sensitivity in a single target mGFR range, X, requires the eGFR range to be wider than X. However, to ensure the eGFR is a unique identifier of sampling time, any neighbouring eGFR ranges must be adjusted, and will thus be narrower. Although using an eGFR range wider than X obtains a high sensitivity in X, the sensitivity in neighbouring ranges is decreased and the specificity in X is decreased. Figure 5 visualises the overlap induced when target sensitivity is applied to each individual range. The overlap is significant, which demonstrates the sensitivity would significantly decrease if unique ranges were attempted. For example, achieving a target sensitivity of 0.9 in the 50–70 mGFR range requires an eGFR range of 35–85, a range that encompasses half of each neighbouring mGFR range. Even for lower target sensitivities significant overlap between ranges is observed.

The analysis was repeated for the four ranges, suggested by McMeekin *et al.* (2022) as opposed to the five ranges specified in the BNMS 2018 guidelines [1,7]. It would be reasonable to assume decreasing the number of ranges may improve the sensitivity in individual windows as these will be wider; however, this was not observed. The variability in eGFR is too high and the sensitivity achieved is still insufficient with four ranges (Fig. 2).

In this dataset applying the BNMS guideline ranges directly to eGFR for the prediction of sampling time would have resulted in 112 studies out of 282 with an incorrectly predicted sampling time-point (40%). Of those incorrectly predicted most of these samples would have been predicted to an adjacent sampling time-point with six studies (2.1%) predicted incorrectly to two sampling time-points away. The increase in uncertainty resulting from incorrect sample time is relatively small [7]. Additionally, the adoption of BNMS 2018 guidelines has been slow in part due to the practical complexities of the number of different time-points.

There was insufficient data in our sample to comment on low GFR results (mGFR < 25 mL/min/1.73 m^2^). Previous work by Hutton *et al.* [8] determined a threshold eGFR of <40 mL/min/1.73 m^2^ to accurately predict patients with mGFR < 25 mL/min/1.73 m^2^, these patients should have a later single-sample time-point [12] or slope-intercept GFR, if a 1-day protocol is preferred [2]. Further work is needed to investigate low GFR results and this would benefit from a multi-centre approach to increase the sample size.

This work shows that although a mathematical optimum sample time-point for single-sample mGFR exists, serum creatinine eGFR is not sufficiently accurate to predict the time-point prior to measurement. Based on this work we suggest a single time-point is used for the majority of GFR patients (mGFR > 25 mL/min/1.73 m^2^). This would enable simpler implementation of the single-sample technique and is warranted as we have demonstrated that eGFR is unable to predict the mGFR values with sufficient accuracy for the selection of optimum single-sample timing. The RMS_err_ for different sample times is shown in Fig. 3 of McMeekin *et al.* (2022) where they demonstrated a ‘wide range of sample times for an acceptably accurate single-sample GFR result’ [7].

### Conclusion

To conclude, the eGFR has high variability resulting in low sensitivity for the prediction of mGFR. This makes eGFR an inappropriate tool for the prediction of optimum sampling time for single-sample mGFR studies over multiple ranges. Where improved accuracy is required, for example at low mGFR (<25 mL/min/1.73 m^2^), a wide eGFR range, as compared to the target mGFR range, should be used to ensure a high sensitivity of prediction. It is the recommendation of this work that two ranges should be applied. The low GFR threshold proposed by Hutton *et al.* (eGFR < 40 mL/min/1.73 m^2^) should be used to ensure all low GFR studies are captured and a single sampling time-point should be selected for all other studies (eGFR > 40 mL/min/1.73 m^2^) due to the poor predictive capability of eGFR.

## Acknowledgements

L.B. is supported by an Integrated Clinical Academic Programme – Healthcare Science Bridging Scheme funded by Health Education England (HEE) South East. The views expressed are those of the authors and not necessarily those of HEE.

### Conflicts of interest

There are no conflicts of interest.
